# Prevalence of ARGs in poultry-associated *E. coli* in Zhejiang Province, China: A genotypic survey

**DOI:** 10.1016/j.isci.2026.116177

**Published:** 2026-06-11

**Authors:** Afeng Wang, Liu Xu, Yuliang Sun, Jiajun Wang, Yu Li, Junhong Sun, Keyu Chen, Yingying Guo, Yanfeng Yi, Yutong Li, Xinyuan Xu, Shengwen Shao, Xinhua Qiang, Jie He, Hongchang Zhou, Yibin Lin, Xiang Wang

**Affiliations:** 1 School of Medicine, Huzhou University, Huzhou 313000, China; 2 School of Science, Huzhou University, Huzhou 313000, China; 3 Infection Disease Department, First People’s Hospital Affiliated to Huzhou University, Huzhou University, Huzhou 313000, China; 4 School of Life, Huzhou University, Huzhou 313000, China; 5 School of Life and Health Sciences, Huzhou College, Huzhou 313000, China; 6 Clinical Laboratory, First People’s Hospital Affiliated to Huzhou University, Huzhou University, Huzhou 313000, China; 7 Key Laboratory for Precise Prevention and Control of Major Chronic Diseases, Huzhou University, Huzhou 31300, China; 8 Zhejiang Key Laboratory of Zero Magnetic Medicine, Affiliated Hangzhou First People’s Hospital, School of Medicine, Westlake University, Hangzhou, Zhejiang, China

**Keywords:** medical surveillance, veterinary medicine, environmental science, microbiology, animal health management, food safety

## Abstract

Antimicrobial resistance (AMR) in food animals is a pressing global One Health concern. Poultry farming in China’s Yangtze River Delta, exemplified by Huzhou City, involves substantial antibiotic use, yet the resultant resistance burden requires characterization. This study quantified clinically relevant antibiotic resistance genes (ARGs) in *E. coli* from poultry farms across Zhejiang Province, China. We found that all 106 isolates harbored at least one ARG, with 99% exhibiting multidrug resistance. Notably, detections of last-resort resistance genes (e.g., *mcr*) were made. Isolates from the major hub, Huzhou, carried a higher ARG burden than those from other regions, and mobile genetic elements were associated with ARG co-occurrence. The findings suggest the presence of antibiotic selection pressure within this system and a concerning burden of ARGs in poultry-associated *E. coli*, with potential implications for environmental and public health. This work highlights the urgent need for integrated interventions, including antibiotic stewardship and surveillance, to mitigate zoonotic AMR risks.

## Highlights

• 99% of poultry *E. coli* were multidrug-resistant, resisting ∼1/3 of antibiotics
• Carbapenemase/optrA genes found in poultry *E. coli* pose a grave clinical threat
• Huzhou is an ARG hotspot driven by mobile genes and co-selection
• Urgent One Health action on antibiotics, genomics, biosecurity vital to curb zoonotic AMR

## Graphical abstract

**Figure:**
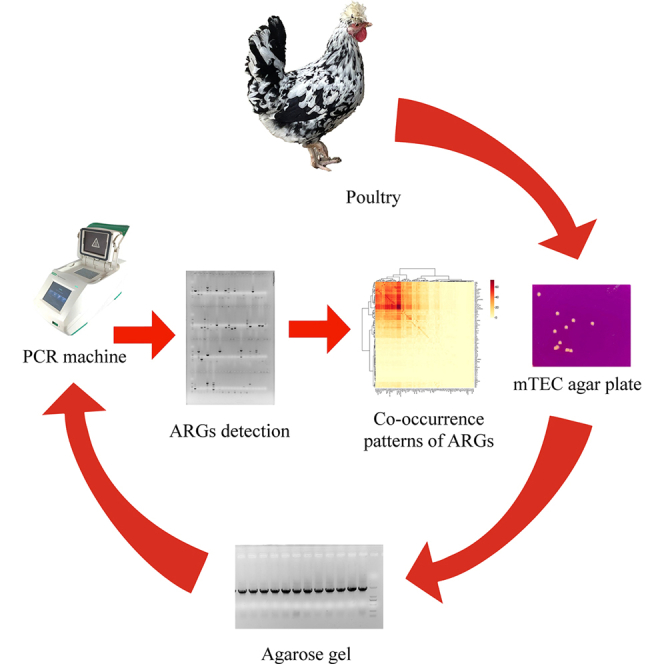

## Introduction

The emergence and global dissemination of antimicrobial resistance (AMR) pose a critical threat to public health and food safety, primarily due to the rampant use of antibiotics in agriculture and medicine, which accelerates the evolution and spread of resistant microorganisms, leading to untreatable infections and heightened mortality rates worldwide.^1^^,^^2^ This crisis is exacerbated by antibiotic resistance genes (ARGs) in zoonotic pathogens that act as key vectors for resistance transmission through the food chain, particularly from poultry to humans, as antibiotic residues and resistant bacteria in farm environments contaminate meat, eggs, and other products, facilitating direct or indirect human exposure.^3^^,^^4^ Environmental antimicrobial-resistant bacteria, often used as indicators of AMR burden, frequently harbor diverse ARGs, including those conferring resistance to critically important antibiotics like beta-lactams and fluoroquinolones.^5^^,^^6^ These genes can transfer to human pathogenic bacteria, such as *Salmonella* or *Klebsiella*, via mobile genetic elements (MGEs) like plasmids, integrons, and transposons, enabling horizontal gene exchange that bypasses species barriers and amplifies resistance in human clinical settings.^7^^,^^8^ The zoonotic transmission of AMR from poultry reservoirs, driven by intensive farming practices and global trade, undermines infection control efforts, increases healthcare costs, and necessitates urgent integrated One Health approaches to mitigate this cross-species spread and safeguard global antimicrobial efficacy.^9^^,^^10^^,^^11^

China’s status as the global leader in poultry production introduces immense complexity to the critical challenge of managing AMR within its intensive farming systems.^12^^,^^13^^,^^14^ These large-scale operations, essential for meeting domestic and international demand, often rely heavily on antimicrobials, creating environments conducive to the emergence and spread of resistance. Within this national context, the Yangtze River Delta region, a powerhouse of the Chinese economy, emerges as a significant focal point for AMR concerns.^15^^,^^16^^,^^17^ Notably, Huzhou City stands out as a major hub within this region, concentrating substantial poultry production activities. The antibiotic usage density in livestock farming across the Delta is exceptionally high, encompassing practices like routine prophylactic administration and prolonged sub-therapeutic dosing. This extensive and often indiscriminate use of antimicrobials exerts intense selective pressure on bacterial populations in poultry reservoirs. This selective pressure acts as a potent driver, significantly accelerating the proliferation, persistence, and horizontal transfer of ARGs among microbes, effectively expanding the environmental resistome.^18^ However, a critical and more focused gap persists: high-resolution, coordinated surveillance data detailing the prevalence, diversity, and co-occurrence dynamics of a broad panel of clinically critical ARGs harbored by *Escherichia coli* in key poultry production hubs like Huzhou City remains limited. Such data are essential for accurate measurement of current public health risks, tracing transmission routes from agricultural sources to humans and ecosystems, and designing evidence-based, targeted interventions needed to combat the growing AMR threat emerging from this critical agricultural region.

*E. coli* serves as an exceptionally suitable target bacterium for investigating the distribution of ARGs in poultry for several compelling reasons.^19^ As a ubiquitous commensal inhabitant of the avian gastrointestinal tract, *E. coli* is readily isolated from poultry samples, providing consistent and ample material for analysis.^20^ More critically, *E. coli* plays a dual role as both a significant pathogen causing various infections in poultry and humans and a vital reservoir for ARGs.^21^ This bacterium demonstrates a remarkable capacity to acquire, harbor, and disseminate a diverse array of resistance determinants, frequently located on highly MGEs like plasmids and integrons.^21^ The ARG profile within poultry *E. coli* isolates functions as a valuable sentinel, reflecting the selective pressure exerted by antibiotic use in the farming environment and indicating the potential reservoir of resistance genes available for horizontal transfer to other bacteria, including zoonotic pathogens. This poses a significant public health risk recognized by international health authorities like the World Health Organization (WHO). Studying *E. coli* thus provides crucial insights into the overall burden and dissemination dynamics of antibiotic resistance within the poultry production system in Huzhou.

This study aims to address this knowledge gap by systematically characterizing the prevalence, diversity, and co-occurrence patterns of clinically relevant ARGs in *E. coli* isolates from commercial poultry farms in Huzhou and surrounding areas of Zhejiang Province. We focus on 92 ARGs spanning 15 major antibiotic classes, prioritized by WHO for their clinical significance,^22^ and employ co-occurrence network analysis to elucidate resistance gene interaction patterns. This focused, in-depth resistome profiling of a major production hub aims to provide data crucial for local risk assessment and targeted intervention strategies within the One Health framework.

Linking ARG detection to spatial and ecological risk models, we provide findings that will inform local antibiotic stewardship policies, guide surveillance priorities for high-risk resistance genes (e.g., carbapenemases), and support One Health strategies to mitigate AMR transmission from poultry production systems to humans.^9^

## Results

### Sample collection and *E. coli* isolation

**Figure 1:**
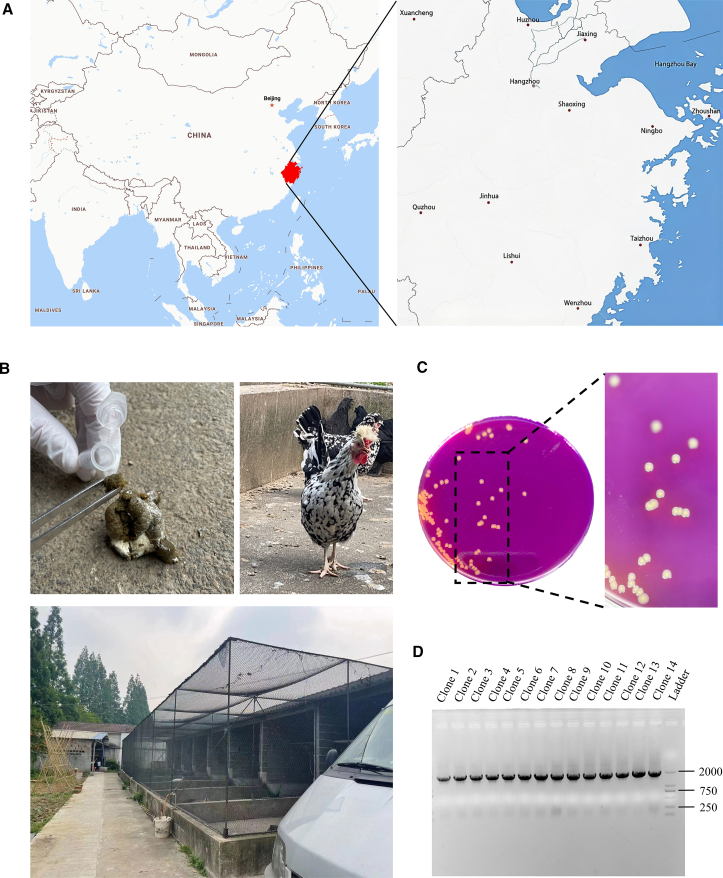
Poultry fecal sampling locations and *E. coli* detection in Huzhou and other cities in Zhejiang Province (A) Map showing poultry fecal sampling sites in Huzhou. (B) Representative field photograph of a poultry fecal sampling location. (C) *E. coli* colonies isolated from poultry fecal samples using the mTEC culture method. (D) Analysis of PCR products by 1% agarose gel electrophoresis.

A total of 106 fecal samples were collected from a selection of poultry farms located in Huzhou City and several other cities within Zhejiang Province, including Hangzhou, Lishui, Ningbo, and Quzhou (Figures 1A and 1B and Table 1). Sample collection took place over a period spanning from May to August 2023. The majority of samples (75/106) originated specifically from farms in Huzhou City, collected across three distinct dates: 25 samples on May 21, 2023 (P1–P25), 25 samples on June 17, 2023 (P26–P50), and 25 samples on July 3, 2023 (P51–P75) (Table 1). An additional 10 samples were collected from Hangzhou farms on July 29, 2023 (P76–P85), and 8 samples were collected from Lishui farms on August 15, 2023 (P86–P93) (Table 1). Samples collected from Ningbo totaled 7 (P94–P100), obtained on August 26, 2023. Six samples were collected from Quzhou farms on August 11, 2023 (P101–P106). Following collection, all 106 samples underwent successful bacterial isolation using selective mTEC agar (Figure 1C). Subsequent PCR analysis confirmed the presence of *E. coli* in every sample (Figure 1D), yielding a total of 106 distinct *E. coli* isolates suitable for further characterization and analysis. The geographic distribution of the collected samples across Zhejiang Province is illustrated in Figure 1A.

**Table 1:** Samples collected from Huzhou and other cities in Zhejiang province

No.	Sites	Date
P1	Huzhou	5/21/2023
P2	Huzhou	5/21/2023
P3	Huzhou	5/21/2023
P4	Huzhou	5/21/2023
P5	Huzhou	5/21/2023
P6	Huzhou	5/21/2023
P7	Huzhou	5/21/2023
P8	Huzhou	5/21/2023
P9	Huzhou	5/21/2023
P10	Huzhou	5/21/2023
P11	Huzhou	5/21/2023
P12	Huzhou	5/21/2023
P13	Huzhou	5/21/2023
P14	Huzhou	5/21/2023
P15	Huzhou	5/21/2023
P16	Huzhou	5/21/2023
P17	Huzhou	5/21/2023
P18	Huzhou	5/21/2023
P19	Huzhou	5/21/2023
P20	Huzhou	5/21/2023
P21	Huzhou	5/21/2023
P22	Huzhou	5/21/2023
P23	Huzhou	5/21/2023
P24	Huzhou	5/21/2023
P25	Huzhou	5/21/2023
P26	Huzhou	6/17/2023
P27	Huzhou	6/17/2023
P28	Huzhou	6/17/2023
P29	Huzhou	6/17/2023
P30	Huzhou	6/17/2023
P31	Huzhou	6/17/2023
P32	Huzhou	6/17/2023
P33	Huzhou	6/17/2023
P34	Huzhou	6/17/2023
P35	Huzhou	6/17/2023
P36	Huzhou	6/17/2023
P37	Huzhou	6/17/2023
P38	Huzhou	6/17/2023
P39	Huzhou	6/17/2023
P40	Huzhou	6/17/2023
P41	Huzhou	6/17/2023
P42	Huzhou	6/17/2023
P43	Huzhou	6/17/2023
P44	Huzhou	6/17/2023
P45	Huzhou	6/17/2023
P46	Huzhou	6/17/2023
P47	Huzhou	6/17/2023
P48	Huzhou	6/17/2023
P49	Huzhou	6/17/2023
P50	Huzhou	6/17/2023
P51	Huzhou	7/3/2023
P52	Huzhou	7/3/2023
P53	Huzhou	7/3/2023
P54	Huzhou	7/3/2023
P55	Huzhou	7/3/2023
P56	Huzhou	7/3/2023
P57	Huzhou	7/3/2023
P58	Huzhou	7/3/2023
P59	Huzhou	7/3/2023
P60	Huzhou	7/3/2023
P61	Huzhou	7/3/2023
P62	Huzhou	7/3/2023
P63	Huzhou	7/3/2023
P64	Huzhou	7/3/2023
P65	Huzhou	7/3/2023
P66	Huzhou	7/3/2023
P67	Huzhou	7/3/2023
P68	Huzhou	7/3/2023
P69	Huzhou	7/3/2023
P70	Huzhou	7/3/2023
P71	Huzhou	7/3/2023
P72	Huzhou	7/3/2023
P73	Huzhou	7/3/2023
P74	Huzhou	7/3/2023
P75	Huzhou	7/3/2023
P76	Hangzhou	7/29/2023
P77	Hangzhou	7/29/2023
P78	Hangzhou	7/29/2023
P79	Hangzhou	7/29/2023
P80	Hangzhou	7/29/2023
P81	Hangzhou	7/29/2023
P82	Hangzhou	7/29/2023
P83	Hangzhou	7/29/2023
P84	Hangzhou	7/29/2023
P85	Hangzhou	7/29/2023
P86	Lishui	8/15/2023
P87	Lishui	8/15/2023
P88	Lishui	8/15/2023
P89	Lishui	8/15/2023
P90	Lishui	8/15/2023
P91	Lishui	8/15/2023
P92	Lishui	8/15/2023
P93	Lishui	8/15/2023
P94	Ningbo	8/26/2023
P95	Ningbo	8/26/2023
P96	Ningbo	8/26/2023
P97	Ningbo	8/26/2023
P98	Ningbo	8/26/2023
P99	Ningbo	8/26/2023
P100	Ningbo	8/26/2023
P101	Quzhou	8/11/2023
P102	Quzhou	8/11/2023
P103	Quzhou	8/11/2023
P104	Quzhou	8/11/2023
P105	Quzhou	8/11/2023
P106	Quzhou	8/11/2023

### ARGs detection overview

**Figure 2:**
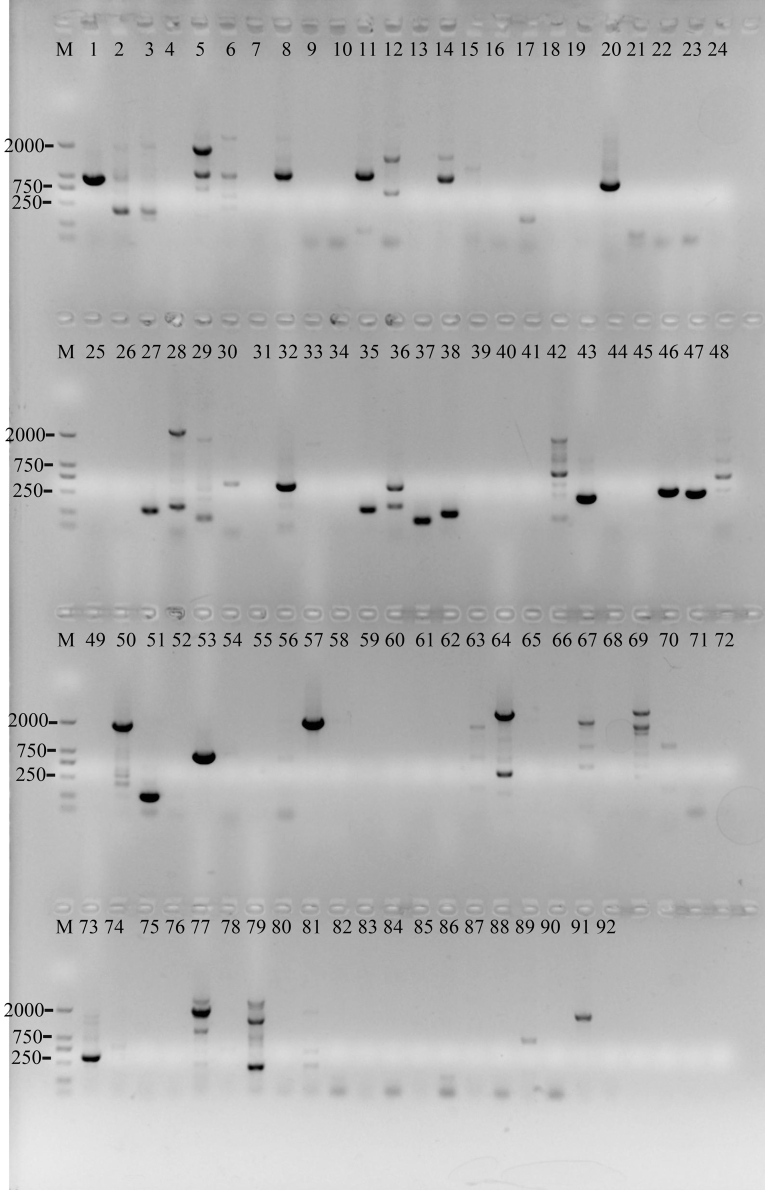
PCR-based antibiotic resistance gene fingerprint patterns of *E. coli* strain isolated from sample P60 The figure displays amplification results for 92 genetic markers associated with AMR. Each lane represents a specific PCR target. 1: β-lactam TEM group. 2–5: β-lactam CTX-M group. 6–10: β-lactam ACC, DHA, FOX, VEB, SHV groups. 11–12: β-lactam CMY group. 13–19: β-lactam IMP, SMP, VIM, OXA, BIC, KPC, NDM groups. 20–21: chloramphenicol (*cmlA*, *catA1*). 22–26: colistin (*mcr*-1, *mcr*-2, *mcr*-3, *mcr*-4, *mcr*-5). 27: florfenicol (*floR*). 28–30: gentamicin (*aac(3)-IV*, *ant(2″)-I*, *aac(3)-II*). 31: linezolid (*optrA*). 32–34: neomycin (*aph(3′)-III-I* variants). 35–45: quinolones (*gyrA Salmonella*, *parC Salmonella*, *gyrA E. coli*, *parC E. coli*, *qnrA*, *qnrB*, *qnrC*, *qnrD*, *qnrS*, *aac(6′)-Ib-cr*, *qepA*). 46–49: streptomycin (*strA*, *strB*, *aadA2*, *aadE*). 50–52: sulfamethoxazole (*sul1*, *sul2*, *sul3*). 53–72: tetracyclines (*tetA*, *tetB*, *tetC*, *tetD*, *tetE*, *tetG*, *tetH*, *tetK*, *tetL*, *tetM*, *tetO*, *tetS*, *tetT*, *tetW*, *tetZ*, *tet31*, *tet32*, *tet33*, *tet34*, *tet39*). 73–75: glycopeptides (*vanA*, *vanB*, *vanX*). 76–80: macrolides (*ermA*, *ermB*, *ermC*, *ermF*). 81–88: streptogramins (*vat*, *vatB*, *vatD*, *vatE*, *vgaA*, *vgaB*, *vgbA*, *vgbB*). 89–92: MRSA-associated markers (*spa*, *mecA*, *mecC*, *pvl*). M: DNA ladder.

The comprehensive molecular screening of 106 *E. coli* isolates from poultry farms across Zhejiang Province revealed a complex landscape of AMR determinants (Table 2). Genomic DNA extracted via phenol-chloroform purification demonstrated high purity (A260/A280 ratios consistently >1.8) and integrity, enabling reliable amplification of 92 clinically significant ARGs representing all major antibiotic classes. PCR analysis using primer sets curated by the Technical University of Denmark’s National Food Institute detected resistance genes in 100% of isolates, with striking heterogeneity in both prevalence and distribution patterns across geographical regions (Figure 2).

**Table 2:** Prevalence and abundance of targeted antibiotic resistance genes

Class	Gene	Prevalence (% of isolates)					
		Huzhou	Hangzhou	Lishui	Ningbo	Quzhou	Total
betalactams	*bla*_TEM_	52	0	12.5	57.14	16.67	42.45
	*bla*_CTX-M_	62.67	10	0	28.57	50	50
	*bla*_CTX-M-1_	58.67	0	37.5	57.14	83.33	52.83
	*bla*_CTX-M-2_	1.33	0	0	0	0	0.94
	*bla*_CTX-M-9_	73.33	20	25	85.71	66.67	65.09
	*bla*_ACC-1_	45.33	10	62.5	28.57	0	39.62
	*bla*_DHA-1_	5.33	0	0	0	0	3.77
	*bla*_FOX_	69.33	10	37.5	42.86	16.67	56.6
	*bla*_VEB-1_	8	0	25	0	0	7.55
	*bla*_SHV_	8	0	12.5	14.29	33.33	9.43
	*bla*_CMY-1_	76	10	25	71.43	66.67	65.09
	*bla*_CMY-2_	66.67	30	37.5	85.71	50	61.32
	*bla*_IMP_	1.33	0	0	0	0	0.94
	*bla*_SMP_	56	40	12.5	57.14	16.67	49.06
	*bla*_VIM_	30.67	40	0	28.57	0	27.36
	*bla*_OXA-48_	1.33	0	0	0	0	0.94
	*bla*_BIC_	28	10	0	42.86	0	23.58
	*bla*_KPC_	1.33	10	0	0	0	1.89
	*bla*_NDM_	6.67	0	0	0	0	4.72
Chloramphenicol	*cmlA*	74.67	60	25	71.43	66.67	68.87
	*catA1*	1.33	0	0	0	0	0.94
Colistin	*mcr-1*	0	0	0	14.29	0	0.94
	*mcr-2*	0	0	0	0	0	0
	*mcr-3*	1.33	0	0	0	0	0.94
	*mcr-4*	0	0	0	0	0	0
	*mcr-5*	2.67	0	0	0	0	1.89
Florphenicol	*floR*	53.33	50	50	85.71	50	54.72
Gentamicin	*aac(3)-IV*	82.67	70	37.5	57.14	100	77.36
	*ant(2″)-I*	68	50	50	71.43	100	66.98
	*aac(3)-II*	20	10	0	0	16.67	16.04
Linezolid	*optrA*	20	50	37.5	28.57	0	23.58
Neomycin	*aph*(3*′*)-III	98.67	100	100	100	100	99.06
	*aph*(3*′*)-II	21.33	10	50	85.71	33.33	27.36
	*aph*(3*′*)-I	30.67	50	37.5	28.57	0	31.13
Quinolones	*gyrA Salmonella*	92	30	62.5	71.43	83.33	82.08
	*parC Salmonella*	73.33	0	25	42.86	66.67	60.38
	*gyrA E. coli*	98.67	90	87.5	100	100	97.17
	*parC E. coli*	97.33	90	87.5	100	100	96.23
	*qnrA*	2.67	0	0	0	0	1.89
	*qnrB*	6.67	30	12.5	14.29	0	9.43
	*qnrC*	0	0	25	28.57	0	3.77
	*qnrD*	58.67	30	62.5	71.43	33.33	55.66
	*qnrS*	33.33	0	25	42.86	50	31.13
	*aac(6′) Ib-cr*	34.67	30	0	0	16.67	28.3
	*qepA*	1.33	0	12.5	14.29	0	2.83
Streptomycin	*strA*	56	30	75	85.71	50	56.6
	*strB*	52	30	62.5	85.71	50	52.83
	*aadA*2	60	20	12.5	42.86	50	50.94
	*aadE*	4	0	0	0	0	2.83
Sulfamethoxazole	*sul1*	69.33	50	12.5	71.43	50	62.26
	*sul2*	70.67	30	25	71.43	50	62.26
	*sul3*	20	0	12.5	0	0	15.09
Tetracycline	*tetA*	62.67	10	25	71.43	83.33	56.6
	*tetB*	13.33	0	0	28.57	0	11.32
	*tetC*	14.67	20	12.5	42.86	16.67	16.98
	*tetD*	10.67	0	12.5	28.57	33.33	12.26
	*tetE*	73.33	10	25	57.14	66.67	62.26
	*tetG*	8	20	0	14.29	16.67	9.43
	*tet(H)*	4	0	0	0	0	2.83
	*tet(K)*	13.33	10	0	14.29	16.67	12.26
	*tet(L)*	5.33	30	37.5	0	16.67	10.38
	*tet(M)*	40	80	62.5	42.86	16.67	44.34
	*tet(O)*	20	20	37.5	57.14	0	22.64
	*tet(S)*	73.33	40	0	57.14	50	62.26
	*tet(T)*	0	0	0	0	0	0
	*tet(W)*	2.67	10	0	0	0	2.83
	*tet(Z)*	50.67	0	0	14.29	16.67	37.74
	*tet(31)*	4	0	0	0	0	2.83
	*tet(32)*	42.67	0	0	14.29	50	33.96
	*tet(33)*	4	0	0	0	0	2.83
	*tet(34)*	2.67	0	0	0	0	1.89
	*tet(39)*	10.67	10	0	0	16.67	9.43
Glycopeptides	*vanA*	76	30	37.5	57.14	66.67	66.98
	*vanB*	20	10	12.5	0	50	18.87
	*vanX*	1.33	0	0	0	0	0.94
Macrolides	*ermA*	2.67	10	0	0	0	2.83
	*ermB*	64	60	75	28.57	83.33	63.21
	*ermC*	30.67	0	0	0	16.67	22.64
	*ermE*	60	30	12.5	42.86	83.33	53.77
	*ermF*	0	0	0	0	0	0
Streptogramins	*vat*	25.33	70	62.5	28.57	16.67	32.08
	*vatB*	1.33	0	0	0	0	0.94
	*vatD*	2.67	0	25	0	0	3.77
	*vatE*	18.67	0	25	14.29	16.67	16.98
	*vgaA*	2.67	10	0	14.29	16.67	4.72
	*vgaB*	2.67	30	62.5	28.57	16.67	12.26
	*vgbA*	4	0	25	14.29	0	5.66
	*vgbB*	1.33	0	12.5	14.29	0	2.83
MRSA	*spa*	33.33	30	12.5	28.57	33.33	31.13
	*mecA*	1.33	0	0	0	0	0.94
	*mecC*	62.67	0	0	42.86	83.33	51.89
	*pvl*	2.67	0	0	0	0	1.89

Beta-lactamases dominated the resistome, particularly plasmid-mediated AmpC enzymes (*bla*_CMY-1_: 65.09%; *bla*_CMY-2_: 61.32%) and extended-spectrum beta-lactamases (ESBLs). The *bla*_CTX-M_ family exhibited notable diversity: *bla*_CTX-M-9_ group emerged as the most prevalent ESBL (65.09% overall), followed by *bla*_CTX-M-1_ group (52.83%). A degree of regional variation was observed. *bla*_CTX-M-1_ prevalence ranged from 0% in Hangzhou isolates to 83.33% in Quzhou isolates. Other beta-lactamase classes like *bla*_OXA-48_ (0.94%), *bla*_KPC_ (1.89%), and *bla*_NDM_ (4.72%) showed minimal detection. Metallo-beta-lactamases (MBLs) showed moderate circulation mainly in Huzhou isolates, with *bla*_VIM_ detected in 27.36% and *bla*_IMP_ in 0.94%.

Tetracycline resistance genes demonstrated extraordinary diversity with 19 variants detected. *tet(S)* (62.26%), *tetA* (56.60%), and *tetE* (62.26%) were highly prevalent, while *tet(32)* (33.96%) and *tet(M)* (44.34%) also circulated widely. Intriguingly, *tet(Z)* (37.74%) was prevalent despite being less commonly reported in poultry. In contrast, clinically critical colistin resistance (*mcr*) genes remain scarce. Specifically, only *mcr-1* (0.94%), *mcr-3* (0.94%), and *mcr-5* (1.89%) were found, primarily in isolates from Huzhou and Ningbo.

Aminoglycoside-modifying enzymes were near-ubiquitous: *aph(3′)-III* (neomycin resistance) was detected in 99.06% of isolates, while *aac(3)-IV* (gentamicin resistance) reached 77.36% prevalence. Streptomycin resistance genes *strA* (56.60%) and *strB* (52.83%) co-occurred frequently. Sulfonamide resistance genes *sul1* (62.26%) and *sul2* (62.26%) were equally prevalent, with *sul*3 (15.09%) less frequent. Phenicol resistance was primarily mediated by *cmlA* (68.87%) and *floR* (54.72%).

Glycopeptide resistance marker *vanA* was detected in 66.98% of isolates overall, peaking at 76% in Huzhou, though *vanB* (18.87%) and *vanX* (0.94%) were less common. Macrolide resistance was primarily driven by *ermB* (63.21%) and *ermE* (53.77%). Quinolone resistance showed high prevalence of chromosomal mutations (*gyrA E. coli*: 97.17%; *parC E. coli*: 96.23%) while plasmid-mediated *qnr* genes followed the order *qnrD* (55.66%) > *qnrS* (31.13%) > *qnrB* (9.43%).

Geographical heterogeneity was pronounced. Huzhou isolates consistently exhibited the highest ARG burdens (e.g., *bla*_CTX-M-9_: 73.33%; *vanA*: 76%), while Hangzhou isolates displayed lower prevalence for most genes (e.g., *bla*_CTX-M-9_: 20%; *vanA*: 30%). No isolates carried *mcr-2*, *mcr-4*, or *tet(T)*, highlighting the absence of these high-threat resistance determinants in this ecosystem. The pervasive co-occurrence of multiple ARG classes foreshadowed the high multidrug resistance rates detailed in subsequent analyses.

### Prevalence of clinically critical ARGs

The targeted PCR screening of 106 *E. coli* isolates from poultry across Zhejiang Province, utilizing primers validated and provided by the Technical University of Denmark’s National Food Institute, revealed a high prevalence and diversity of genes associated with clinically critical antibiotic resistance. The analysis focused on ARGs deemed critical for human medicine by organizations like the WHO, particularly those conferring resistance to last-resort or widely used therapeutic classes.

Resistance to beta-lactam antibiotics, especially extended-spectrum cephalosporins and carbapenems, was widespread and driven by diverse mechanisms. Genes encoding ESBLs were highly prevalent. The *bla*_CTX-M-9_ was detected in 65.09% of isolates overall, with striking regional variation (85.71% in Ningbo, 73.33% in Huzhou, but only 20% in Hangzhou). The *bla*_CTX-M-1_ group was also common (52.83% overall), reaching 83.33% in Quzhou. AmpC beta-lactamase genes were extremely frequent, particularly the *bla*_CMY-1_ (65.09% overall, 76% in Huzhou) and *bla*_CMY-2_ (61.32% overall, 85.71% in Ningbo). The *bla*_FOX_ group AmpC gene was detected in 56.60% of isolates overall (69.33% in Huzhou). Worryingly, genes associated with carbapenem resistance were present despite lower prevalence. *bla*_VIM_ variants occurred most frequently (27.36% overall; 40% in Hangzhou, 30.67% in Huzhou), followed by *bla*_NDM_ variants (4.72% overall, exclusively in Huzhou at 6.67%), *bla*_OXA-48_ (0.94%), *bla*_KPC_ variants (1.89%), and *bla*_IMP_ variants (0.94%). Metallo-beta-lactamase genes (*bla*_BIC_) were found in 23.58% of isolates.

The critical last-resort antibiotic colistin showed evidence of emerging resistance mediated by plasmid-borne *mcr* genes. While *mcr*-1 was detected in only one isolate overall (0.94%, specifically from Ningbo), *mcr-3* and *mcr-5* were each found in 0.94% and 1.89% of isolates, respectively, predominantly within the Huzhou region (1.33% and 2.67% local prevalence). *mcr-2* and *mcr-4* were not detected in any isolate.

Resistance to fluoroquinolones, vital for treating serious Gram-negative infections, was pervasive and mediated by multiple mechanisms. Mutations in chromosomal topoisomerase genes (*gyrA*, *parC*) were near-ubiquitous in the *E. coli* background (*gyrA E. coli*: 97.17%; *parC E. coli*: 96.23%). Plasmid-mediated quinolone resistance (PMQR) genes were also widespread, particularly *qnrD* (55.66% overall, 58.67% in Huzhou, 71.43% in Ningbo) and *qnrS* (31.13% overall, 33.33% in Huzhou). The *aac(6′) Ib-cr* variant, conferring resistance to both aminoglycosides and fluoroquinolones, was found in 28.30% of isolates (34.67% in Huzhou). *qnrB* was detected in 9.43% of isolates.

Glycopeptide resistance genes, though less common in Gram-negatives, were surprisingly frequent. *vanA*, typically associated with high-level vancomycin resistance in enterococci but increasingly detected in Gram-negatives, was present in a notable 66.98% of isolates overall (76% in Huzhou, 66.67% in Quzhou). *vanB* was found in 18.87% of isolates.

Resistance to macrolides and streptogramins (used against Gram-positives but monitored due to horizontal gene transfer potential) was also notable. The macrolide resistance gene *ermB* was prevalent (63.21% overall, 75% in Lishui, 83.33% in Quzhou), as was *ermE* (53.77% overall, 60% in Huzhou). Among streptogramin resistance genes, *vat* was the most common (32.08% overall, 70% in Hangzhou).

For other critical antibiotic classes, aminoglycoside resistance (e.g., to gentamicin) frequently involved *aac(3)-IV* at 77.36% overall (Quzhou: 100%; Huzhou: 82.67%) and *ant(2″)-I* at 66.98% overall (Quzhou: 100%). The linezolid resistance gene *optrA*, associated with transferable resistance to this important oxazolidinone antibiotic, was detected in 23.58% of isolates overall, with notably high prevalence in Hangzhou (50%) and Lishui (37.5%). Chloramphenicol resistance via *cmlA* was common (68.87% overall), while florfenicol resistance (*floR*) was detected in 54.72% of isolates. Tetracycline resistance genes were ubiquitous, led by *tetA* (56.60%), *tetE* (62.26%), *tetS* (62.26%), and *tet(32)* (33.96%). Sulfonamide resistance genes *sul*1 and *sul2* were each found in 62.26% of isolates.

This targeted screening revealed a high prevalence and diversity of genes associated with clinically critical antibiotic resistance among poultry-derived *E. coli* in Zhejiang Province, including multiple genes conferring resistance to WHO-classified highest priority critically important antimicrobials (HP-CIAs) like colistin, carbapenems, and fluoroquinolones. Regional variations were evident, with Huzhou showing high burdens of many key resistance determinants compared to other areas. The presence of transferable resistance genes like *mcr-3*, *mcr-5*, *optrA*, *qnrD*, *bla*_NDM_, and *bla*_CTX-M_ variants underscores the potential zoonotic reservoir these poultry isolates represent.

### Multi-drug resistance profiles

A notable finding of this study was the widespread occurrence of multidrug resistance (MDR) among the 106 *E. coli* isolates recovered from poultry across Huzhou and other Zhejiang sites. Every single isolate (100%) harbored resistance genes conferring resistance to three or more distinct classes of antimicrobials, satisfying the MDR definition used in this analysis (Tables 3 and S2). The extent of MDR, quantified as the percentage of tested ARGs detected per isolate relative to the total 92-gene panel, revealed a notable variation among sampling locations (Tables 3 and S2). Prevalence rates ranged from 1.08% to 50%, with a mean MDR prevalence of 28.61% ± 10.33% across all isolates. This indicates that, on average, isolates carried resistance determinants for approximately one-third of the clinically relevant ARGs targeted.

**Table 3:** Prevalence of multi-drug resistance in Huzhou and other cities in Zhejiang province

No.	Sites	Prevalence (% of ARG tested)	Mean prevalence (%)
P1	Huzhou	35.8	31.06
P2	Huzhou	32.61	
P3	Huzhou	14.13	
P4	Huzhou	30.43	
P5	Huzhou	30.43	
P6	Huzhou	31.52	
P7	Huzhou	29.35	
P8	Huzhou	23.91	
P9	Huzhou	40.22	
P10	Huzhou	27.17	
P11	Huzhou	36.96	
P12	Huzhou	30.43	
P13	Huzhou	25	
P14	Huzhou	42.39	
P15	Huzhou	32.61	
P16	Huzhou	35.87	
P17	Huzhou	36.96	
P18	Huzhou	39.13	
P19	Huzhou	35.87	
P20	Huzhou	34.78	
P21	Huzhou	43.48	
P22	Huzhou	22.83	
P23	Huzhou	26.09	
P24	Huzhou	20.65	
P25	Huzhou	27.17	
P26	Huzhou	29.35	
P27	Huzhou	21.74	
P28	Huzhou	8.7	
P29	Huzhou	26.09	
P30	Huzhou	29.35	
P31	Huzhou	46.74	
P32	Huzhou	50	
P33	Huzhou	47.83	
P34	Huzhou	16.3	
P35	Huzhou	35.87	
P36	Huzhou	42.39	
P37	Huzhou	28.26	
P38	Huzhou	45.65	
P39	Huzhou	40.22	
P40	Huzhou	30.43	
P41	Huzhou	40.22	
P42	Huzhou	43.48	
P43	Huzhou	34.78	
P44	Huzhou	40.22	
P45	Huzhou	43.48	
P46	Huzhou	31.52	
P47	Huzhou	20.65	
P48	Huzhou	23.91	
P49	Huzhou	25.34	
P50	Huzhou	29.35	
P51	Huzhou	36.96	
P52	Huzhou	33.7	
P53	Huzhou	38.04	
P54	Huzhou	41.3	
P55	Huzhou	41.3	
P56	Huzhou	30.43	
P57	Huzhou	29.35	
P58	Huzhou	5.43	
P59	Huzhou	33.7	
P60	Huzhou	25	
P61	Huzhou	28.26	
P62	Huzhou	33.7	
P63	Huzhou	25	
P64	Huzhou	21.74	
P65	Huzhou	33.7	
P66	Huzhou	15.22	
P67	Huzhou	7.61	
P68	Huzhou	16.3	
P69	Huzhou	34.78	
P70	Huzhou	35.87	
P71	Huzhou	39.13	
P72	Huzhou	29.35	
P73	Huzhou	33.7	
P74	Huzhou	21.74	
P75	Huzhou	20.65	
P76	Hangzhou	10.87	
P77	Hangzhou	11.96	
P78	Hangzhou	20.65	
P79	Hangzhou	39.13	17.83
P80	Hangzhou	13.04	
P81	Hangzhou	11.96	
P82	Hangzhou	16.3	
P83	Hangzhou	2.17	
P84	Hangzhou	26.09	
P85	Hangzhou	26.09	
P86	Lishui	1.09	20.11
P87	Lishui	15.22	
P88	Lishui	18.48	
P89	Lishui	33.7	
P90	Lishui	14.13	
P91	Lishui	17.39	
P92	Lishui	28.26	
P93	Lishui	32.61	
P94	Ningbo	28.26	38.04
P95	Ningbo	13.04	
P96	Ningbo	28.26	
P97	Ningbo	32.61	
P98	Ningbo	29.35	
P99	Ningbo	36.96	
P100	Ningbo	40.22	
P101	Quzhou	35.87	29.81
P102	Quzhou	26.63	
P103	Quzhou	21.74	
P104	Quzhou	21.74	
P105	Quzhou	22.83	
P106	Quzhou	26.09	

Beta-lactam resistance genes were the cornerstone of the observed MDR profiles, exhibiting near ubiquity and comprising the most diverse and frequently detected class. Genes conferring resistance to quinolones (via mutations in *gyrA* and *parC*), tetracyclines, aminoglycosides (particularly *aph(3′)-III* and *aac(3)-IV)*, and sulfonamides (*sul1*, *sul2*) were also exceptionally common and frequently co-occurred within isolates. Macrolide (*ermB*, *ermE*), phenicol (*floR*), and glycopeptide (*vanA*) resistance genes further notably contributed to the complex MDR signatures. The high prevalence of clinically critical resistance determinants was particularly concerning. ESBL genes, primarily within the *bla*_CTX-M_ groups (especially *bla*_CTX-M-1_ and *bla*_CTX-M-9_ groups), were detected in the vast majority of isolates (>80%), underlining a major challenge for beta-lactam therapy. PMQR genes (*qnrD*, *qnrS*, *aac(6′)-Ib-cr*) and genes associated with MRSA (*mecC*, *spA*) were also identified in substantial proportions of isolates, further complicating potential treatment options.

Analysis of the MDR profiles revealed evident co-resistance patterns. Isolates rarely carried resistance determinants for only a single antibiotic class; instead, genes from multiple classes like ESBL genes (*bla*_CTX-M_ groups), *tet* genes (especially *tetA*, *tetE*), sulfonamide resistance genes (*sul1*, *sul2*), aminoglycoside resistance genes (*aph(3′)-III*, *aac(3)-IV*), and PMQR genes (*qnrD*, *qnrS*) were consistently found together, forming a frequent core within the complex MDR backgrounds observed across numerous sites (Table S2). Spatial analysis indicated some heterogeneity in MDR prevalence (Table 3). Sites P41 and P42 in Huzhou exhibited the highest average MDR prevalence (40.22% and 43.48%, respectively), while site P58 showed the lowest (5.43%). Sites in Hangzhou (e.g., P83 at 2.17%) and Lishui (e.g., P86 at 1.09%) also demonstrated lower, though still detectable, MDR prevalence. This variation suggests potential differences in selective pressures or contamination sources between specific farms or geographical clusters within the study region.

The near-universal detection of ARG-defined multidrug resistance profiles, driven by a diverse array of high-prevalence resistance genes across critical antibiotic classes and characterized by extensive co-resistance, highlights a profoundly compromised effectiveness of multiple antimicrobial classes against *E. coli* in the studied poultry populations.

### Co-occurrence patterns and clusters of ARGs

**Figure 3:**
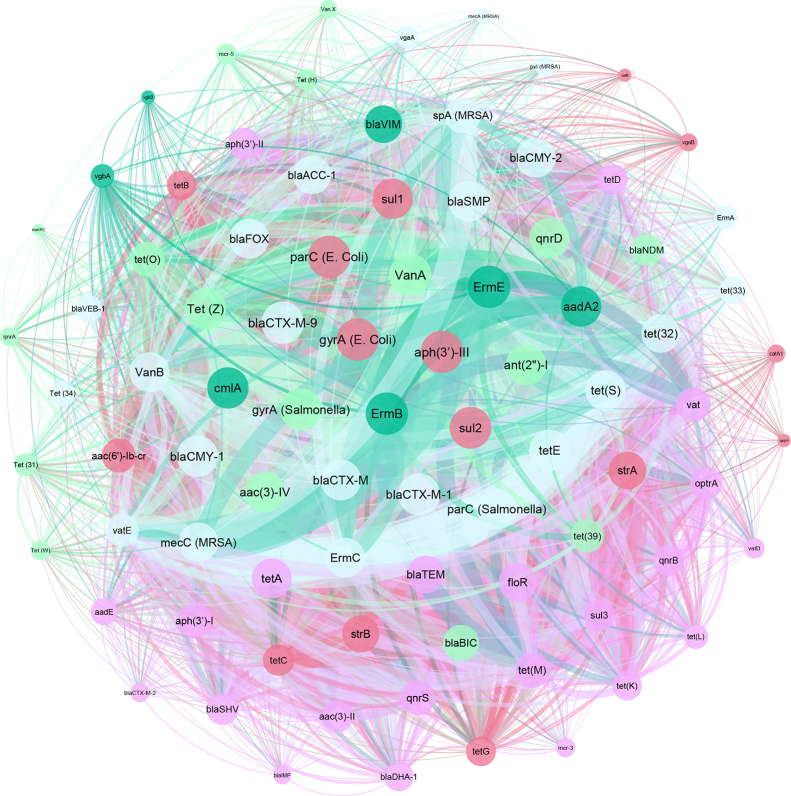
Co-occurrence network of antibiotic resistance genes (ARGs) in poultry fecal microbiome Nodes represent individual ARGs, sized by their co-occurrence rate and colored by resistotype. Edges denote statistically supported co-occurrence relationships (Jaccard Index > 0.65; *p* < 0.001), based on presence/absence profiles across isolates. The network was constructed and visualized using an optimized topological layout in Gephi 0.10.1.

**Figure 4:**
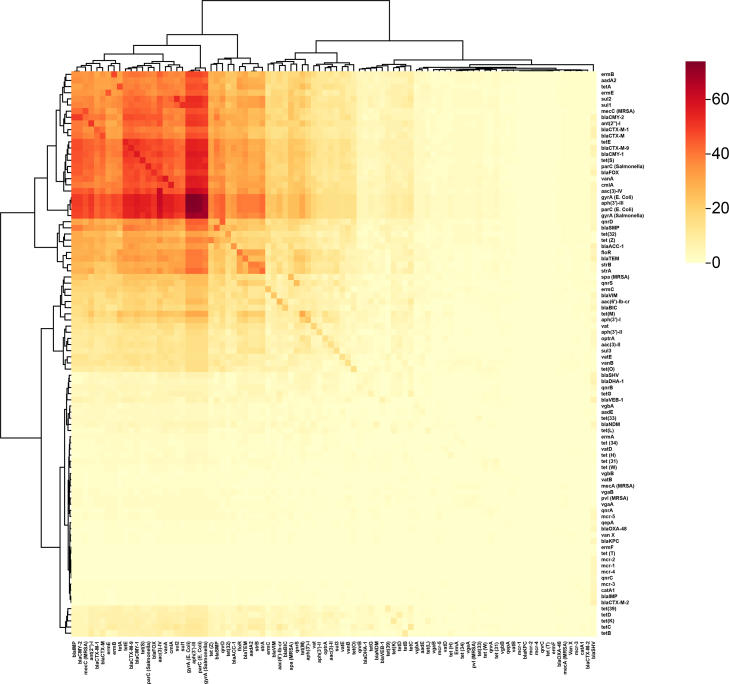
Heatmap of ARG co-occurrence patterns The heatmap illustrates pairwise associations among antibiotic resistance genes (ARGs) based on their co-occurrence across all isolates. Rows and columns represent individual ARGs, and hierarchical clustering dendrograms group ARGs with similar co-occurrence profiles. The color gradient (yellow to dark red) represents the Jaccard similarity index value for each ARG pair, ranging from 0 (no co-occurrence) to 60 (perfect co-occurrence).

The complex interplay of ARGs was systematically investigated through co-occurrence network analysis and hierarchical clustering of the 75 *E. coli* isolates in Huzhou. Co-occurrence networks constructed using the Jaccard similarity index revealed four dominant gene clusters exhibiting strong synergistic persistence (Figure 3). The most robust module comprised *aph(3′)-III*, *gyrA* (*E. coli*), and *parC* (*E. coli*), which co-occurred in 98.4% of isolates carrying any of these genes (Jaccard Index > 0.98). This triad was frequently linked to *gyrA* (*Salmonella*), *aac(3)-IV*, *bla*_CMY-1_, *cmlA* with co-occurrence frequency 93.15%, 83.56%, 76.71%, and 76.71, respectively, suggesting possible co-selection or genetic linkage on shared MGEs, which may facilitate their dissemination as a unit (Figure 4). Notably, *aph(3′)-III* emerged as a critical hub gene, demonstrating a notable association with 85 other ARGs across multiple antibiotic classes (degree centrality = 27), including *sul2* (ρ = 0.72, *p* < 0.001), *bla*_FOX_ (ρ = 0.77, *p* < 0.001), and *sul*1 (ρ = 0.70, *p* < 0.001) (Figure 4).

**Figure 5:**
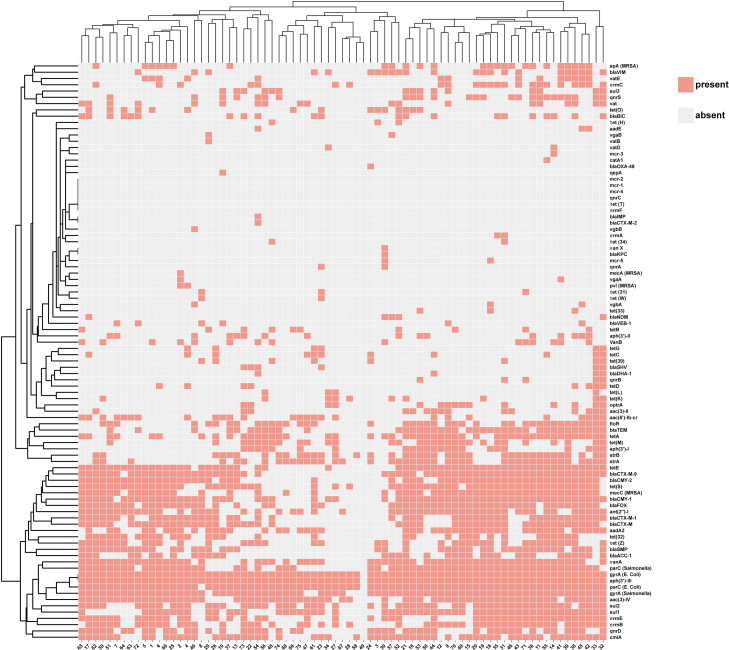
Hierarchical cluster analysis of antibiotic resistance gene presence across *E. coli* samples Heatmap showing the presence (red) or absence (gray) of 92 antibiotic resistance genes (ARGs) (rows) in 75 *E. coli* samples (columns). Analysis identified patterns of gene co-occurrence and sample resistance profile similarity. Rows (genes) were clustered using the simple matching coefficient and Ward linkage; columns (samples) were clustered using Jaccard distance and Ward linkage. The dendrogram to the left represents clustering of genes (rows), while the dendrogram above represents clustering of samples (columns).

Hierarchical cluster analysis was performed on the binary detection data of 92 ARGs across 75 *E*. *coli* samples to identify patterns of co-occurrence among genes and similarities in resistance profiles among samples. Both dimensions, genes (rows) and samples (columns), were clustered using appropriate distance metrics (e.g., Jaccard distance for samples, simple matching coefficient for genes) and the Ward linkage method (Figure 5). The resulting dendrograms reveal distinct patterns. Clustering of the samples identified several subgroups characterized by shared resistance gene combinations. Larger clusters primarily consisted of samples exhibiting high carriage rates of widespread genes like *aph(3*′*)-III* (aminoglycoside resistance) and mutations in *gyrA* (fluoroquinolone resistance). Smaller, tightly clustered groups were detected showing co-enrichment for specific sets of genes; for instance, samples clustered together based on the presence of combinations such as *bla*_CTX-M-9_, *bla*_TEM_, *bla*_CMY-1_, *tetE*, *sul1*, and *aac(3)-IV*. Gene clustering revealed strong functional and likely genetic linkage patterns. Genes conferring resistance to the same antibiotic class frequently clustered together. Notably, genes encoding ESBLs (e.g., *bla*_CTX-M-9_, *bla*_CTX-M-1_, *bla*_CTX-M_) formed a prominent cluster, highlighting a notable ESBL burden. Genes associated with aminoglycoside resistance (e.g., *aph(3′)-III*, *ant(2″)-I*, *strA*, *strB*) clustered tightly, suggesting potential co-acquisition on shared genetic elements. A distinct cluster included *tet* genes, particularly *tetE* and *tetA*, indicative of prevalent tetracycline resistance mechanisms. Genes rarely detected (e.g., *mcr* variants, *bla*_KPC_, *bla*_NDM_, *bla*_OXA-48_) form separate, isolated branches or clustered loosely, reflecting their sporadic occurrence. The analysis underscores the dominance of β-lactam (especially ESBLs), aminoglycoside, tetracycline, and sulfonamide resistance mechanisms within this collection of *E. coli* isolates, while also identifying specific co-occurrence patterns within samples and functional groups within the resistome. The presence of defined sample clusters suggests potential clonal spread or shared selective pressures within specific subsets of isolates.

## Discussion

It is important to note that PCR detection indicates genetic potential and not confirmed phenotypic expression. The PCR method is a widely validated approach for detecting ARGs^23^^,^^24^^,^^25^ and was employed in this study to analyze ARG carriage in *E. coli* isolates from poultry in Zhejiang Province, with a focus on Huzhou City. Results reveal a dire landscape of resistance prevalence, diversity, and complexity among poultry-associated *E. coli*, highlighting a substantial burden of antimicrobial resistance genes (ARGs) in poultry-associated *E. coli*, which may have relevance for animal and public health within the One Health paradigm.

While large-scale surveillance studies in China have established the high prevalence of ESBLs and AmpC genes in poultry-associated *E. coli*,^26^^,^^27^^,^^28^ this study provides a focused, in-depth resistome profile of a critical poultry production hub—Huzhou City in the Yangtze River Delta. Our contribution lies in: (1) applying a comprehensive, clinically-focused 92-ARG panel to quantify the burden of high-threat resistance determinants (e.g., *mcr, optrA*, *bla*_NDM_) in this specific system; (2) employing co-occurrence network and hierarchical clustering analyses to reveal gene interaction patterns and potential clonal clusters; and (3) linking high ARG burdens and MDR profiles to specific geographical locations within the province, thereby identifying potential hotspots for targeted surveillance.

Our findings unequivocally demonstrate that poultry-derived *E. coli* in Zhejiang, particularly in Huzhou, serve as a major reservoir for diverse ARGs. Detection of at least one ARG in all isolates, which span all major antibiotic classes tested, highlights a baseline saturation of resistance determinants. Resistome characterization revealed extreme diversity (e.g., 19 distinct tetracycline resistance genes) and high prevalence across gene classes. Notably, beta-lactam resistance genes dominated, particularly plasmid-mediated AmpC genes (*bla*_CMY-1_, *bla*_CMY-2_, *bla*_FOX_) and ESBLs (*bla*_CTX-M-9_, *bla*_CTX-M-1_), reflecting intense selective pressure from beta-lactam use in poultry production. These trends mirror findings from large-scale Chinese surveillance studies, which also emphasize ESBL and AmpC dominance in poultry-associated *E. coli*.^26^^,^^27^^,^^28^ Our study aligns with recent work in eastern China, but the prevalence of certain high-threat genes, such as *vanA* (66.98% overall) and *optrA* (23.58% overall), appears notably high in our Huzhou-focused collection compared to some broader regional averages, potentially indicating local intensification of selective pressures or unique resistance dissemination dynamics.

Alarmingly, *vanA* prevalence reached 66.98% overall (76% in Huzhou), a concerning expansion of glycopeptide resistance in Gram-negative bacteria typically associated with Gram-positive pathogens like enterococci.^29^ This finding highlights risks of zoonotic transmission and treatment failures due to MGEs.^30^ Similarly, the detection of carbapenemase genes (e.g., *bla*NDM in 4.72% of isolates) in this poultry reservoir exceeds rates reported in recent *Salmonella* studies from eastern China,^27^ raising concerns about interspecies gene transfer in this hotspot. Carbapenems, critical last-resort antibiotics, face erosion as resistance mechanisms spread within poultry reservoirs linked to human populations via food chains and environmental contamination.^31^^,^^32^^,^^33^ The potential for carbapenemase-producing bacteria to undermine this final line of defense constitutes a medical crisis scenario.^34^^,^^35^

While acknowledging PCR-based detection’s limitations in inferring phenotypic resistance, our findings align with prior studies demonstrating strong concordance between certain ARGs (e.g., *bla*_CTX-M_, *mcr-1*) and phenotypic resistance in poultry-associated *E. coli*.^36^^,^^37^ Future studies integrating antimicrobial susceptibility testing (AST) with genomic surveillance are critical to validate these associations and refine risk assessments.

The convergence of high-threat resistance in a prevalent agricultural system represents a dangerous erosion of global antimicrobial defenses. This necessitates urgent, coordinated surveillance, stewardship, and containment interventions.^2^ While plasmid-mediated colistin resistance genes (*mcr-1*, *-3*, *-5*) remain quantitatively low (0.94%–1.89%), their detection confirms the ongoing emergence and stable persistence of resistance mechanisms for this vital last-resort antibiotic.^38^^,^^39^ Geographical concentration around Huzhou and Ningbo suggests potential local hotspots for transmission or selective pressures, demanding focused surveillance.

Equally alarming is the high prevalence of the transferable oxazolidinone resistance gene *optrA* (23.58% overall; 50% in Hangzhou). Oxazolidinones (e.g., linezolid) are critical for treating multidrug-resistant Gram-positive infections (e.g., vancomycin-resistant *Enterococcus*, methicillin-resistant *Staphylococcus aureus*).^40^ The high frequency of *optrA*, carried on MGEs, potentially compromises this drug class’ efficacy and increases horizontal transmission risks to human pathogens.^40^^,^^41^^,^^42^^,^^43^

Fluoroquinolone resistance was nearly ubiquitous, with chromosomal mutations in *gyrA* and *parC* and high prevalence of PMQR genes. This synergistic resistance undermines fluoroquinolone efficacy across multiple drug classes, rendering treatment options unreliable.^44^

The universal detection of ARG-defined multidrug resistance profiles in 106 isolates (100% prevalence) and the high average MDR prevalence (28.61%) underscore an extreme resistance burden. This level of MDR surpasses rates frequently reported in global poultry studies,^45^^,^^46^^,^^47^^,^^48^ highlighting the severity of the challenge in this intensive production system. Isolates carried resistance determinants for ∼33% of 92 screened ARGs. Core MDR patterns included ESBLs (*bla*_CTX-M_), AmpCs (*bla*_CMY-1/2_, *bla*_Fox_), tetracyclines (*tetA*, *tetE*, *tetS*), sulfonamides (*sul1*, *sul2*), aminoglycosides (*aph(3′)-III*, *aac(3)-IV*), PMQR (*qnrD*), and glycopeptides (*vanA*). Co-resistance rendered multiple antibiotic classes ineffective, leaving few therapeutic options.

Geographical heterogeneity in ARG prevalence and MDR burden were observed among the sampled locations. Isolates from Huzhou generally exhibited higher prevalences of several key ARGs (e.g., *bla*_CTX-M-9_, *vanA*, *aac(3)-IV*, *cmlA*, *bla*_Fox_, and *bla*_CMY-1_) alongside elevated MDR rates compared to those from Hangzhou or Lishui. Intensive antibiotic selection pressure in Huzhou’s poultry sector, potentially linked to prophylactic/metaphylactic use, warrants investigation. Farms with highest MDR burdens (e.g., P9, P14, P21, P39, P41, P42, P45) require urgent intervention.

Co-occurrence networks and hierarchical clustering revealed resistance stabilization via co-selection and MGEs. The *aph(3′)-III*-*gyrA*-*parC* triad and its associations with *aac(3)-IV*, *bla*_CMY-1_, *cmlA*, *sul1*, and *sul2* are consistent with possible physical linkage or co-selection on shared MGEs (e.g., plasmids and integrons). This allows cross-class resistance maintenance even without direct selection pressure. *aph(3′)-III*’s near-universal presence (99.06%) and high centrality in resistance networks underscores its role as a resistance hub. Consistent with recent reports on resistance gene co-occurrence,^49^^,^^50^^,^^51^^,^^52^ this study elucidates resistance gene interaction patterns in a key agricultural region through intuitive visualization, thereby shedding light on the development of complex resistance phenotypes and revealing potential avenues for blocking gene transfer.

Clustering analysis confirmed resistance genes’ functional class co-localization (e.g., ESBLs, aminoglycosides, and tetracyclines) and distinct strain/subpopulation groupings, suggesting clonal spread or horizontal gene transfer. This aligns with IncHI1 plasmid-mediated resistance patterns observed globally.^53^^,^^54^

The near-universal MDR phenotype (99%) and prevalence of high-priority critically important antimicrobials (HP-CIAs), such as colistin, carbapenems, and fluoroquinolones, pose a direct zoonotic threat. Resistant bacteria carrying *mcr-3*/*5*, *optrA*, *qnrD*, *bla*_NDM_, and *bla*_CTX-M_ variants could disseminate via waste contamination, food chains, and farmworker exposure, potentially jeopardizing human health and contributing to treatment failures for common infections (e.g., fluoroquinolone-resistant urinary tract infections [UTIs]) or life-threatening carbapenem-resistant systemic infections, increasing morbidity, mortality, and healthcare costs.^55^^,^^56^^,^^57^^,^^58^ Huzhou’s extreme MDR burden highlights vulnerabilities at the animal-human interface, demanding urgent One Health interventions. Urgent, multifaceted interventions within a One Health framework are essential to mitigate this threat. These should include functional validation of ARGs (e.g., conjugation assays) to determine their actual transmission risks in poultry production systems.

### Limitations of the study

While providing a comprehensive snapshot of ARG carriage in *E. coli* from the studied farms, our study has several limitations. First, our reliance on PCR-based detection of ARGs precludes direct assessment of phenotypic resistance. Future work must integrate AST to link gene presence with resistance phenotypes, enabling robust risk stratification. Second, the absence of conjugation assays prevents definitive conclusions about the horizontal transferability of the detected ARGs. Future studies should incorporate mating experiments to evaluate transmission potential. Third, the cross-sectional, convenience sampling design, focused on Huzhou, limits the generalizability of the prevalence estimates to the broader poultry population in Zhejiang Province. The lack of detailed, farm-level antibiotic usage data also hinders direct correlation between specific practices and the observed resistance patterns. Finally, the use of selective mTEC agar may have biased the recovered *E. coli* population. Future investigations should prioritize longitudinal, structured sampling paired with antibiotic use surveys and employ complementary methods (e.g., metagenomics and WGS) to provide deeper insights into resistance dynamics and transmission.

## Resource availability

### Lead contact

Further information and requests for resources should be directed to the lead contact, Xiang Wang (wangxiang2021@zju.edu.cn).

### Materials availability

Bacterial isolates generated in this study are available from the lead contact upon reasonable request. This article does not generate new unique reagents.

### Data and code availability

• The antibiotic resistance gene (ARG) presence/absence matrix for all 106 *E. coli* isolates has been included as Table S3 and is publicly available as of the date of publication. Accession codes are listed in the key resources table.
• This article does not report the original code.
• Any additional information required to reanalyze the data reported in this article is available from the lead contact upon request.

## Acknowledgments

This work was funded by the 10.13039/501100001809National Natural Science Foundation of China (82472291 and 82372329); 10.13039/501100004731Zhejiang Provincial Natural Science Foundation of China (LTGD24C010001); Zhejiang Province Key Research and Development Plan Project (2023C03048); Key Project of Health Science and Technology Plan of Hangzhou (TD2023009); and 10.13039/501100009988Huzhou Municipal Science and Technology Bureau (2025YZ33, 2024GZ74, 2024GZ81, and 2021GZ62). The authors thank all of the people who help us to collect avian fecal samples.

## Author contributions

Y.L. and X.W. designed research; A.W., X.X., J.W., Y.L., L.X., J.S., K.C., and Y.G. performed research; S.S., H.Z., J.H., Y.S., and Y.L. performed the bioinformatics analysis; X.Q., Y.Y., J.W., and L.X. provided materials; Y.L., X.W., H.Z., and Y.S. wrote the article.

## Declaration of interests

The authors declare no competing interests.

## Declaration of generative AI and AI-assisted technologies in the writing process

During the preparation of this work the authors used Deepseek-R1 for language refinement. After using this tool, the authors reviewed and edited the content as needed and take full responsibility for the content of the published article.

## STAR★Methods

### Key resources table

**Table:** 

REAGENT or RESOURCE	SOURCE	IDENTIFIER
**Bacterial and virus strains**		

*E. coli* isolates as defined in Table 1	This paper	N/A

**Biological samples**		

Fresh fecal samples (n=75)	This paper; poultry farms in Huzhou, Zhejiang Province, China	N/A
Fresh fecal samples (n=10)	This paper; poultry farms in Hangzhou, Zhejiang Province, China	N/A
Fresh fecal samples (n=8)	This paper; poultry farms in Lishui, Zhejiang Province, China	N/A
Fresh fecal samples (n=7)	This paper; poultry farms in Ningbo, Zhejiang Province, China	N/A
Fresh fecal samples (n=6)	This paper; poultry farms in Quzhou, Zhejiang Province, China	N/A

**Chemicals, peptides, and recombinant proteins**		

buffered peptone water (BPW)	BBI	Cat#B681010-0250
mTEC ChromoSelect Agar	Millipore	Cat#90924
Luria broth (LB) Broth	Sangon Biotech	Cat#A507002
Lysozyme Solution	BBI	Cat#B686024
Ezup Column Bacteria Genomic DNA Purification Kit	Sangon Biotech	Cat#B518255
Agarose	BBI	Cat#A620014
Taq PCR Master Mix (2×, with Red Dye)	BBI	Cat#B639297
GoldView nucleic acid stains	Yeasen	Cat#10201ES03
DNA Marker (200∼2000 bp)	BBI	Cat#B600339

**Deposited data**		

ARG presence/absence matrix for all *E. coli* isolates	This paper	Table S3

**Oligonucleotides**		

Primer for *E. coli uidA* gene detection Forward: GGATCTTCCAGAGATATGT TACGTCCTGTAGAAACCCCA	This paper; Designed based on known *E. coli uidA* sequence (NCBI Reference Sequence: NC_000913.3)	N/A
Primer for *E. coli uidA* gene detection Reverse: CTGCCGTTCGACGATTCAT TGTTTGCCTCCCTGCT	This paper; Designed based on known *E. coli uidA* sequence (NCBI Reference Sequence: NC_000913.3)	N/A
Primers for 92 antibiotic resistance genes screening, see Table S1	This paper; National Food Institute, Technical University of Denmark (DTU Food)	N/A

**Software and algorithms**		

Gephi	Bastian et al. (The Gephi Consortium)	Version 0.10.1; https://gephi.org
Python	Python Software Foundation	Version 3.13; https://www.python.org
GraphPad Prism	GraphPad Software	Version 10.5; https://www.graphpad.com

### Experimental model and study participant details

#### Ethical statement

This study solely involved the collection of avian fecal samples from the environment. No experimental procedures were performed on live animals or human subjects. Therefore, ethical approval for animal experimentation or human subject research was not required. The study protocol complied with local regulations regarding environmental sample collection.

#### Study system and sample allocation

The study utilized a system of *E. coli* bacteria that was isolated from poultry fecal samples. The source hosts for these bacterial isolates were commercial poultry, obtained from farms located in Zhejiang Province, China. Regarding sample size and allocation, a total of 106 fresh fecal samples were aseptically collected from various poultry farms. The distribution of these samples based on geographic location was as follows: 75 samples were collected from Huzhou City, 10 from Hangzhou City, 8 from Lishui City, 7 from Ningbo City, and 6 from Quzhou City. The sampling activities were conducted during the period spanning May to August 2023. From each individual fecal sample, one distinct *E. coli* isolate was successfully purified and confirmed, culminating in a final collection of 106 unique *E. coli* isolates available for subsequent analysis.

#### Bacterial strains and culture conditions

Presumptive *E. coli* isolates were cultured from fecal samples following initial enrichment in Buffered Peptone Water (BPW) and subsequent plating on selective mTEC Agar. Characteristic colonies were purified on LB agar and definitively confirmed as *E. coli* via PCR targeting the species-specific *uidA* gene. All confirmed isolates were preserved long-term in glycerol-enriched LB broth at -80°C.

### Method details

#### Sample collection and processing

A total of 106 fresh fecal samples were aseptically collected from poultry farms located in major poultry-producing regions across Zhejiang Province, China, during the period spanning May to August 2023. Targeted sampling focused on Huzhou City (representing a major poultry-producing hub in the region), with additional convenience samples collected from the cities of Hangzhou, Lishui, Ningbo, and Quzhou to provide preliminary geographical context within the province. This was a cross-sectional, focused survey rather than a randomized, population-representative study. The specific sample distribution by location and collection date showed that Huzhou City had 75 samples collected across three dates, with 25 samples gathered on May 21, 2023 (samples P1-P25), 25 samples on June 17, 2023 (P26-P50), and 25 samples on July 3, 2023 (P51-P75). Hangzhou City contributed 10 samples collected on July 29, 2023 (P76-P85), while Lishui City provided 8 samples collected on August 15, 2023 (P86-P93). Ningbo City yielded 7 samples collected on August 26, 2023 (P94-P100), and Quzhou City supplied 6 samples collected on August 11, 2023 (P101-P106). Immediately after collection, the samples were placed in sterile, leak-proof containers, stored on ice, and transported to the laboratory within 8 hours. Upon arrival, the samples were processed promptly for bacterial isolation.

Farm-level antibiotic usage data were unavailable for this study due to privacy restrictions and the retrospective nature of sample collection. Future studies should integrate longitudinal surveillance of antibiotic stewardship practices with genomic resistance monitoring.

#### *E. coli* isolation and confirmation

Each fecal sample (approximately 1 g) was homogenized in 9 ml of sterile buffered peptone water (BPW) and incubated at 37°C for 24 hours to promote enrichment. A 10 μl loopful of the enriched suspension was then streaked onto selective chromogenic mTEC agar (modified Tryptone Bile X-Glucuronide Agar) plates.^59^ Plates were incubated aerobically at 44.5 ± 0.5°C for 24 ± 2 hours. Following incubation, presumptive *E. coli* colonies (typically appearing as yellow to yellow-brown) were selected for purification. Pure isolates were obtained by streaking representative colonies onto non-selective Luria broth (LB) agar plates and incubating at 37°C for 18-24 hours. One distinct *E. coli*-like isolate per original fecal sample was purified and preserved. All presumptive pure isolates underwent definitive confirmation using polymerase chain reaction (PCR). Genomic DNA was extracted from overnight broth cultures grown in LB using a boiling lysis method (resuspended colony in 50 μl sterile ddH_2_O, boiled at 100°C for 10 min, cooled on ice, centrifuged, supernatant used as template). PCR amplification targeting a conserved *E. coli*-specific genetic marker (e.g., *uidA* gene) was performed with the forward primer GGATCTTCCAGAGATATGTTACGTCCTGTAGAAACCCCA and the reverse primer CTGCCGTTCGACGATTCATTGTTTGCCTCCCTGCT. Amplification products were analyzed by agarose gel electrophoresis (1%) to confirm the expected amplicon size. A total of 106 isolates, one per collected fecal sample, were confirmed as *E*. *coli*.

It is acknowledged that the use of mTEC agar at 44.5°C may selectively enrich for certain *E. coli* populations, potentially excluding strains with reduced growth at this temperature or those lacking β-glucuronidase activity (e.g., some extraintestinal pathogenic *E. coli* serotypes). Future studies could consider supplementing with alternative cultivation methods (e.g., non-selective media or broader temperature ranges) to enhance detection comprehensiveness.^59^^,^^60^

#### Genomic DNA extraction for ARG screening

High-quality genomic DNA (gDNA) was extracted from overnight broth cultures (LB, 37°C) of all 106 confirmed *E. coli* isolates using the phenol-chloroform isoamyl alcohol (PCI) extraction method, as previously described with modifications.^61^ Briefly, cell pellets were lysed using enzymatic (lysozyme) and chemical (SDS) lysis steps. Proteins were precipitated and removed, followed by extraction with PCI and precipitation of DNA using ice-cold ethanol or isopropanol. The concentration and purity of the extracted gDNA were rigorously assessed using a NanoDrop spectrophotometer. Samples consistently demonstrated high purity, indicated by A260/A280 ratios >1.8. The integrity of the DNA was further confirmed by visualization on an agarose gel to ensure minimal shearing. Purified gDNA was diluted to a standardized working concentration (e.g., 25-50 ng/μl) in nuclease-free water and stored at -20°C for subsequent PCR analysis.

#### Detection of antibiotic resistance genes (ARGs)

A comprehensive panel of 92 clinically significant antibiotic resistance genes (ARGs) was selected for screening. This panel targeted all major classes of antibiotics, including: beta-lactams (*bla*_TEM_, *bla*_CTX-M_, *bla*_CTX-M-1_, *bla*_CTX-M-2_, *bla*_CTX-M-9_, *bla*_ACC-1_, *bla*_DHA-1_, *bla*_FOX_, *bla*_VEB-1_, *bla*_SHV_, *bla*_CMY-1_, *bla*_CMY-2_, *bla*_IMP_, *bla*_SMP_, *bla*_VIM_, *bla*_OXA-48_, *bla*_BIC_, *bla*_KPC_, *bla*_NDM_), Chloramphenicol (*cmlA*, *catA1*), colistin (*mcr*-1 to *mcr*-5), florphenicol (*floR*), gentamicin (*aac(3)-I*, *vant(2")-I*, *aac(3)-II*), linezolid (*optrA*), neomycin (*aph(3')-III*, *aph(3')-II*, *aph(3')-I*), quinolones (*gyrA Salmonella*, *parC Salmonella*, *gyrA E. coli*, *parC E. coli*, *qnrA*, *qnrB*, *qnrC*, *qnrD*, *qnrS*, *aac(6') Ib-cr*, *qepA*), streptomycin (*strA*, *strB*, *aadA2*, *aadE*), sulfamethoxazole (*sul*1, *sul*2, *sul*3), tetracycline (*tetA*, *tetB*, *tetC*, *tetD*, *tetE*, *tetG*, *tet(H)*, *tet(K)*, *tet(L)*, *tet(M)*, *tet(O)*, *tet(S)*, *tet(T)*, *tet(W)*, *tet(Z)*, *tet(31)*, *tet(32)*, *tet(33)*, *tet(34)*, *tet(39)*), glycopeptides (*vanA*, *vanB*, *vanX*), macrolides (*ermA*, *ermB*, *ermC*, *ermE*, *ermF*), streptogramins (*vat*, *vatB*, *vatD*, *vatE*, *vgaA*, *vgaB*, *vgbA*, *vgbB*), MRSA (*SPA*, *mecA*, *mecC*, *pvl*).

Oligonucleotide primers for all 92 target ARGs were sourced from validated primer sets curated and published by the National Food Institute, Technical University of Denmark (DTU Food) (Table S1). Each PCR reaction (typically 25 μl) contained: PCR buffer, dNTPs (200 μM each), MgCl_2_ (concentration optimized per primer set), forward and reverse primers (0.2-0.5 μM each), Taq DNA polymerase (1.25 U), and template DNA (2-5 μl). Thermocycling conditions generally consisted of: an initial denaturation (95°C for 5 min); followed by 35-40 cycles of denaturation (95°C for 20 sec), annealing (optimized primer-specific temperature, e.g., 50-60°C for 20 sec), and extension (72°C for 60 sec per kb); with a final extension (72°C for 10 min). Annealing temperatures and extension times were optimized for each individual primer pair prior to high-throughput screening to ensure specificity and efficiency. PCR amplification products were resolved on 1.0% agarose gels stained with GoldView nucleic acid stains and visualized under UV light. The presence of an amplicon of the expected size was recorded as a positive result for that specific ARG in that isolate. Positive controls (gDNA from reference strains known to harbor specific ARGs) and negative controls (reaction mixture with nuclease-free water instead of template DNA) were included in each PCR run to ensure assay specificity and detect contamination. Each isolate-ARG combination was tested at least once. Ambiguous results were confirmed by repeat PCR.

#### Analysis of results and statistics

ARG prevalence calculation: The prevalence (%) of each ARG was calculated as the number of isolates testing positive for the gene divided by the total number of isolates analyzed (n=106), multiplied by 100. Prevalence calculations were also performed for subsets defined by geographical origin (Huzhou, n=75; Hangzhou, n=10; Lishui, n=8; Ningbo, n=7; Quzhou, n=6).

Multidrug resistance (MDR) definition and quantification: MDR was defined as the presence of ARGs conferring resistance to three or more distinct classes of antimicrobials. To quantify the overall resistance potential per isolate, the percentage of ARGs detected per isolate (i.e., number of ARGs detected in an isolate divided by the total 92 ARGs screened, multiplied by 100) was calculated and defined as the “MDR prevalence” for that isolate. Average MDR prevalence per region was calculated from individual isolate MDR prevalence values.

Co-occurrence analysis: Association patterns between different ARGs were evaluated using co-occurrence network analysis. The Jaccard similarity index was calculated for each pairwise combination of ARGs based on their binary (presence/absence) profile across all isolates. Significant associations (Jaccard Index > 0.65; p-value < 0.001) were used to construct an undirected co-occurrence network using specialized software (Gephi 0.10.1). Hierarchical clustering (using binary distance metric and average linkage) of the entire binary ARG presence/absence matrix (isolates x antibiotic resistance genes) was performed to identify clusters of functionally related genes (modules) and clusters of isolates with similar ARG profiles (clades).

Resistotype Definition: Based on the results of hierarchical clustering and observed frequent co-occurrence patterns, distinct resistotypes (R-types) were defined by characteristic combinations of specific, clinically critical ARGs.

Statistical Testing: Regional differences in ARG prevalence and R-type distribution were assessed for statistical significance using Chi-square (χ^2^) tests with a significance level of p < 0.05. Specific correlations mentioned within the co-occurrence network results were likely assessed using correlation coefficients (ρ) calculated during network analysis and reported with corresponding p-values.

We note that PCR-based detection of ARGs indicates genetic potential for resistance but does not equate to phenotypic resistance. Functional validation (e.g., via antibiotic susceptibility testing or gene expression analysis) was not performed in this study due to resource constraints. However, the near-universal co-occurrence of ARGs with chromosomal mutations (e.g., *gyrA*/*parC*) and mobile genetic elements (e.g., plasmids) suggest a high likelihood of functional resistance in these isolates.^62^

### Quantification and statistical analysis

Data were analyzed using statistical software (Python, GraphPad Prism). Co-occurrence networks of antibiotic resistance genes (ARGs) were constructed in Gephi based on significant pairwise associations (Jaccard Index > 0.65; p < 0.001). Regional differences in ARG prevalence and resistance type (R-type) distribution were assessed using Chi-square tests (p < 0.05). Specific correlations within the network are reported with correlation coefficients (ρ) and p-values.

### Additional resources

No new websites or forums were created for this study. No supplementary resources were employed in this study.

## Supplemental information

Table S1. The primers used in ARGs screening The primers used in this study were sourced from Technical University of Denmark, National Food Institute.

Table S2. Prevalence of multi-drug resistance in Huzhou and other cities in Zhejiang province

Table S3. Antibiotic resistance gene (ARG) presence/absence matrix for all 106 *E. coli* isolates
